# Supplementary material for: Transcriptome Analysis Reveals Critical Genes and Pathways in Carbon Metabolism and Ribosome Biogenesis in Poplar Fertilized with Glutamine
Source: Int J Mol Sci. 2022 Sep 2;23(17):9998. doi: 10.3390/ijms23179998 (PMC9456319; doi:10.3390/ijms23179998)
Supplement: Supplementary file 1 [file ijms-23-09998-s001.zip › ijms-1888059-supplementary.pdf]

Table S1. Primer sequences used for qRT-PCR analysis

Gene id	Gene function	Gene name	Primer sequence-FW	Primer sequence-RV
POPTR_005G079200v3	aspartate aminotransferase	*AspAT1*	GCAGAGGTTGAAGGGTTTGG	TGTAGGGTGGTCAGAGAAGC
POPTR_006G241600v3	aspartate aminotransferase 3	*AspAT4*	GTTGCAGGCAGGGTTGAAAG	GGCTTCAAAGAGTTTCTGGCG
POPTR_006G260200v3	aspartate aminotransferase 5	*AspAT5*	TGCACGTGGTATGGAGCTTT	TCGGGCAATCCTTTTCAGCT
POPTR_014G143300v3	aspartate aminotransferase 1	*AspAT7*	AGCAATCCTCCTGTTCAT	CAGCCATTACCTTCACTTC
POPTR_018G022200v3	aspartate aminotransferase 5	*AspAT9*	TCCTCCTGTTCATGGGGCTA	CCTCCCAGCCATCATTTCCA
POPTR_018G082500v3	aspartate aminotransferase 2	*AspAT10*	CACTGGCTTGAACACTGAACA	AAAAGATCCCCAGAAGCGGA
POPTR_005G172400v3	nitrate reductase 2	*NIA2.2*	AATGCCGAAGCCTGGTGG	CATGACTCGGCGTCGTTATC
POPTR_012G011700v3	NADH-dependent glutamate synthase 1	*GOGAT2*	GGGAACCACTGGTAACCGAAG	AATCGTGAGTGCACCAACTC
POPTR_006G038400v3	glutamate synthase 1	*GOGAT1*	GTCCAGTCAACGCCACTCTC	CAAGTTTGCTACCTGCGGTT
